# Glow Discharge in a High-Velocity Air Flow: The Role of the Associative Ionization Reactions Involving Excited Atoms

**DOI:** 10.3390/ma12162524

**Published:** 2019-08-08

**Authors:** Ezequiel Cejas, Beatriz Rosa Mancinelli, Leandro Prevosto

**Affiliations:** 1 Grupo de Descargas Eléctricas, Departamento Ingeniería Electromecánica, Facultad Regional Venado Tuerto, Universidad Tecnológica Nacional, Laprida 651, 2600 Venado Tuerto, Santa Fe, Argentina; 2 Grupo de Descargas Eléctricas, Departamento Ingeniería Electromecánica, Facultad Regional Venado Tuerto, Universidad Tecnológica Nacional, Consejo Nacional de Investigaciones Científicas y Técnicas (CONICET), Laprida 651, 2600 Venado Tuerto, Santa Fe, Argentina

**Keywords:** glow discharge, air kinetic scheme, non-equilibrium discharge

## Abstract

A kinetic scheme for non-equilibrium regimes of atmospheric pressure air discharges is developed. A distinctive feature of this model is that it includes associative ionization with the participation of N(^2^D, ^2^P) atoms. The thermal dissociation of vibrationally excited nitrogen molecules and the electronic excitation from all the vibrational levels of the nitrogen molecules are also accounted for. The model is used to simulate the parameters of a glow discharge ignited in a fast longitudinal flow of preheated (*T*_0_ = 1800–2900 K) air. The results adequately describe the dependence of the electric field in the glow discharge on the initial gas temperature. For *T*_0_ = 1800 K, a substantial acceleration in the ionization kinetics of the discharge is found at current densities larger than 3 A/cm^2^, mainly due to the N(^2^P) + O(^3^P) → NO^+^ + e process; being the N(^2^P) atoms produced via quenching of N_2_(A^3^∑_u_^+^) molecules by N(^4^S) atoms. Correspondingly, the reduced electric field noticeably falls because the electron energy (6.2 eV) required for the excitation of the N_2_(A^3^∑_u_^+^) state is considerably lower than the ionization energy (9.27 eV) of the NO molecules. For higher values of *T*_0_, the associative ionization N(^2^D) + O(^3^P) → NO^+^ + e process (with a low–activation barrier of 0.38 eV) becomes also important in the production of charged particles. The N(^2^D) atoms being mainly produced via quenching of N_2_(A^3^∑_u_^+^) molecules by O(^3^P) atoms.

## 1. Introduction

A number of experiments have been reported on non-equilibrium regimes of discharges in atmospheric-pressure air, in particular, glow-type discharges in open ambient air (e.g., [1,2,3,4,5,6,7,8,9,10,11,12]), and in fast longitudinal flows of air [13,14]. In [13,14], the parameters of a low-current glow discharge in a high-velocity flow of preliminary heated air (*T*_0_ = 1800–2900 K) at atmospheric pressure, is investigated. The discharge is ignited between two needle electrodes oriented along the axis of the gas flow directed from the cathode to the anode. High-speed gas flows have been used to provide sufficient cooling of discharges at high pressures [15,16]. If the gas residence time in the discharge is small as compared with the vibrational-translational (V-T) relaxation time, the gas heating is almost suppressed and the gas is in a strongly non-equilibrium state (i.e., characterized by a level of vibrational energy which considerable exceeds its equilibrium value). These regimes, which correspond to relatively high electron number densities, 10^18^–10^19^ m^−3^, and relatively low gas temperatures, 2000–3000 K, are of interest for many practical applications, including plasma decontamination and sterilization, material processing, modification of electromagnetic waves propagation, and plasma aerodynamics (see the recent review [17] and references therein). On the other hand, for discharge conditions such that the gas residence time in the discharge is larger than the V-T relaxation time (e.g., discharges stabilized by natural convection), the air changes to a state close to the thermodynamically equilibrium one, with a higher gas temperature as in low-current arc discharges. 

Several kinetic schemes have been proposed in the literature for modelling atmospheric pressure non-equilibrium low-current air discharges: streamers [18,19], low-current arc and glow discharges at rest or in low-gas flows [8,20,21,22], glow discharges in fast-gas flows [14,23,24], and high-current pulsed discharges (leaders [25,26,27,28,29,30]), in the gas temperature range *T*_g_ = 1000–6000 K. Although there are some differences in the chemical and electron kinetics considered in those works, they all coincide in the fact that a change in the dominant ionization mechanism takes place: from ionization of NO molecules by electron impact e + NO → e + e + NO^+^ at *T*_g_ < 4500 K, to associative ionization in ground–state atomic collisions N(^4^S) + O(^3^P) → e + NO^+^, at higher gas temperatures.

In [31], the importance of the reactions of associative ionization involving excited atoms, N(^2^P) + O(^3^P) → e + NO^+^, was demonstrated in a non-self–sustained glow discharge in atmospheric pressure nitrogen with a small admixture of oxygen. However, the associative ionization reactions involving N(^2^D, ^2^P) metastable atoms, are not routinely considered in air kinetic models [8,14,18,19,20,21,22,23,24,25,26,27,28,29,30].

A kinetic scheme for non-equilibrium regimes of atmospheric pressure air discharges is presented. A distinctive feature of this model is that it includes associative ionization reactions involving excited atoms. The model is used to simulate the parameters of a strongly non-equilibrium discharge in fast longitudinal air flows under the experimental conditions of [13,14]. The paper is organized as follows: Section 2 describes the numerical model, while the results and its discussion are presented in Section 3; Section 4 summarizes the conclusions.

## 2. The Model 

The model incorporates 98 reactions (Table 1) among the following active species for pure air: N_2_(X^1^∑_g_^+^, v), N_2_(A^3^∑_u_^+^), N_2_(B^3^Π_g_), N_2_(a*’*^1^∑_u_^−^), N_2_(C^3^Π_u_), O_2_, NO, N(^4^S), N(^2^D), N(^2^P), O(^3^P), O(^1^D), O(^1^S), NO^+^, N_2_^+^, O_2_^+^, O^+^, O^–^, O_2_^–^, O_3_^–^, and electrons (e). The calculation of the transport and rate coefficients of the electrons is based on finding the electron energy distribution function (EEDF) in terms of the local reduced electric field *E*/*N* (*E* is the electric field strength and *N* the gas number density) by means of the solution of the electron Boltzmann equation in a two-term approximation with the BOLSIG+ code [32]. An estimation of an effective electron temperature *T*_e_ (as two-thirds of the mean-electron energy) is also obtained by means of the BOLSIG+ code. The corresponding cross sections were taken from [33]. Note that the use of the local field approximation for the calculation of electron transport and rate coefficients is justified when the electron energy relaxation time for achieving a steady state EEDF is small compared with the characteristic discharge timescale, and the length of the electron energy relaxation is much smaller than the characteristic discharge radius. Both conditions usually hold under regimes typical of atmospheric pressure molecular plasmas (e.g., [15,34]).

Among the processes of charged particle production, the model incorporates the associative ionization reaction with the participation of excited atoms (R18), which have no activation barrier; and the near-threshold reaction (R16), with a low-activation barrier of 0.38 eV. The efficiency of these processes is determined by the decrease in ionization threshold resulting from the chemical bond energy of the compound molecular ion. The reaction between the N(^2^P) and O(^3^P) atoms was observed in experiments [54]; the rate constant of the decay of the N(^2^P) atoms in the reaction with O(^3^P) was found to be (1.7 ± 0.4) 10^–17^ m^3^/s, with the formation of NO^+^ as a significant reaction channel. This value agrees with estimate [36]. Cross section data for the ionization reaction between the N(^2^D) and O(^3^P) were measured in [39]. Following [25], the rate coefficient of the reaction (R13) was taken from [35]. The calculations are performed using rates for reactions (R22) and (R23) taken from [44].

The coefficients for electron-impact processes are calculated for the mixture N_2_–20%O_2_ (the rate constants for electrons depend on the mixture composition, i.e. on the degree of dissociation of oxygen, only slightly [20,25]). The influence of the deformation of the EEDF (due to superelastic collisions) on the high-threshold electron-impact processes (electronic excitation, dissociation, ionization and dissociative attachment), is accounted for as in [15,20,34]. The increase of the electronic excitation rates (including the electron-impact dissociation) due to the inclusion of excitation from all the vibrational levels of N_2_(X^1^∑_g_^+^, v) (v = 0 to 67, according to [55,56]) is considered as [57,58,59],

(1) $$\;k_{j}\;\left(\mathrm{all}\right)=k_{j}\;\left(v=0\right)\sum_{v=0}^{v_{\max}}\exp\;\left[\frac{\hbar \;\omega }{k_{b}}\left(\frac{1}{T_{e}}\;-\;\frac{1}{T_{v}}\right)\right]$$

*k*_j_(v = 0) is the constant for the *j*th excitation process from vibrational ground-state N_2_(X^1^∑_g_^+^, v = 0) molecules calculated with the BOLSIG+ code, ▽ω is the nitrogen vibrational quantum and *k*_b_ the Boltzmann’s constant. *T*_v_ is the vibrational temperature of the nitrogen molecules related to its mean–vibrational energy as *ε*_v_ = ▽ω/[exp(▽ω/(*k*_b_ *T*_v_)) − 1]. Equation (1) results from the simple assumption of reducing the threshold of the cross sections for the corresponding N_2_(X^1^∑_g_^+^, v = 0) process according to the excitation energy of the reactants [60,61,62] and of a Boltzmann distribution for the populations of N_2_(X^1^∑_g_^+^, v) molecules at *T*_v_. Note that Equation (1) reduces to *k*_j_(all) = v_max_ *k*_j_(v = 0) for *T*_e_ = *T*_v_ [58].

The conditions of vibrational excitation also facilitate the thermal dissociation of the nitrogen. The dependence of the rate of the thermal dissociation of N_2_(X^1^∑_g_^+^, v) on the vibrational temperature *T*_v_ is take into account through the Macheret-Fridman model [63,64], which was found to be the most accurate for nitrogen dissociation under non-equilibrium conditions [65]. The vibrational temperatures of other molecular air components, such as O_2_ and NO, are close to the gas temperature due to a fast V–T relaxation of these species (e.g., [15]).

For the conditions of [13,14], the preheated airflow enters the discharge region with a mean–velocity *u*_0_ ≈ 450 m/s, the discharge length along the flow *L* = 3.5 cm, the discharge radius *R* ≈ 1.6 mm, the gas pressure *p* = 1 atm. The Reynolds number, based on the nozzle diameter (*D* = 1 cm), is 1× 10^4^–2 × 10^4^. For such conditions, the radial transport of particles (and also heat) due to radial inhomogeneities is negligible small as compared to the longitudinal (along the axis) convective transport caused by axial inhomogeneities. The radial diffusion timescale for neutral and charged species may be estimated as *R*^2^/*D*_t_, where the diffusion coefficient *D*_t_ (≈ 0.0045 *u*_0_ *D* [15]) accounts for the gas turbulence effects as the gas flow is likely fully turbulent [66]. (Note that *D*_t_ is several times greater than that corresponding to laminar flows [15].) It follows that the radial diffusion timescale *R*^2^/*D*_t_ is about 1.3 × 10^−4^ s under the analyzed conditions. This value can be compared to the mean-convective transport time of the gas in the discharge region, *τ* ≈ *u*_0_/(2*d*) ≈ 3.9 × 10^−5^ s. The factor 2 in the estimation of *τ*, considers that, on average, the gas is transported along half the length of the discharge [15]. The inequality *R*^2^/*D*_t_ >> *τ* indicates that the transport of particles in the radial direction is negligible as compared to the longitudinal transport caused by convection. See also estimates in [23]. Note that spatially resolved optical measurements of N_2_ C state emission in the conditions of [13,14] show that the radius of the discharge is nearly constant along the discharge axis (i.e., the radial diffusion of electrons from the discharge core is small); thus, supporting the above estimation. In addition, the diffusion timescale of heat may be estimated as *N c*_p_ *R*^2^/λ, where *c*_p_ is the mean–specific heat at constant pressure per heavy particle [67] and *λ* the translational thermal conductivity of heavy particles. For *T*_g_ = 2000–3000 K, *λ* is about 0.1 W/(s m) [68]. The corresponding ratio *N c*_p_ *R*^2^/λ/*τ* is of the order of 100. 

To reveal the influence of the associative ionization with the participation of exited atoms, on the air ionization kinetics in the conditions of [13,14], the balance equations are solved in a local (volume-averaged) approximation [69]; but the longitudinal inhomogeneities caused by convection is also accounted for. This is done by introducing a term (*Y*_i0_ − *Y*_i_)/*τ* [15,70] in the balance equation for each *Y*_i_ plasma quantity; where *Y*_i0_ is the value of the *Y*_i_ quantity at the section where the preheated air enters the discharge column (just after the cathodic part of the discharge).

The balance equations for the plasma particles under the local approximation,

(2) $$\frac{\partial }{\partial t}\left(\left[Y_{i}\right]\right)=\sum_{j}S_{ij}-\frac{\left[Y_{i}\right]-\left[Y_{i0}\right]}{\tau }$$

where [*Y_i_*] is the number density and *S_i j_* is the rate of production of the *Y*_i_ species in the *j*th reaction (negative if the species is destroyed), are solved for the species N_2_(A^3^∑_u_^+^), N_2_(a*’*^1^∑_u_^−^), NO, N(^4^S), N(^2^D), N(^2^P), O(^3^P), O(^1^D), O(^1^S), N_2_^+^, O_2_^+^, O^+^, O^–^, O_2_^–^, O_3_^–^, and electrons. The rate to produce the N_2_(A^3^∑_u_^+^) state by cascading is assumed to be equal to the sum of the rates for the production by electron impact of the N_2_(B^3^Π_g_) and N_2_(C^3^Π_u_) states [71,72]. Estimates show [71] that this simplification causes a negligible error in the rate of production of the N_2_(A^3^∑_u_^+^) state. By making this assumption, the populations of the N_2_(B^3^Π_g_) and N_2_(C^3^Π_u_) states do not need to be computed directly. The density of the dominating sort of positive ions NO^+^ (all primary ions formed in ionizing collisions of electrons with air components other than NO convert quickly to NO^+^ under the conditions considered) is obtained from the condition of quasi-neutrality. The density of the dominant species N_2_(X^1^∑_g_^+^, v) is obtained from the constancy of the pressure, while the density of O_2_ is calculated by the condition of conservation of N and O nuclei. The balance equations for the plasma particles are coupled with the balance equations describing the mean–vibrational energy of the nitrogen molecules and the mean-kinetic energy of the gas,

(3) $$\frac{\partial }{\partial \;t}\;\left(\left[N_{2}\left(X\right)\right]\;\varepsilon_{v}\right)=\eta_{V}\;\sigma E^{2}-\left[N_{2}\left(X\right)\right]\;\frac{\varepsilon_{v}-\varepsilon_{v}\left(T_{g}\right)}{\tau_{\mathrm{VT}}}-\left[N_{2}{\left(X\right)}_{0}\right]\;\frac{\varepsilon_{v}-\varepsilon_{v}\left(T_{0}\right)}{\tau }$$

(4) $$\frac{\partial }{\partial \;t}\left(T_{g}\;\sum_{i}\left[Y_{i}\right]\;c_{p\;i}\right)=Q_{R}+\eta_{T}\;\sigma E^{2}+\left[N_{2}\left(X\right)\right]\;\frac{\varepsilon_{v}-\varepsilon_{v}\left(T_{g}\right)}{\tau_{\mathrm{VT}}}-\sum_{i}\left[Y_{i}{}_{0}\right]\;c_{p\;i}\frac{T_{g}-T_{0}}{\tau }$$

*η*_V_ and *η*_T_ are the fractions of electron energy transferred to gas heating and to the vibrational excitation of N_2_(X^1^∑_g_^+^, v) molecules, and *σ* is the electrical conductivity of the plasma. ε_v_(*T*_g_) is the equilibrium value of the mean–vibrational energy of the nitrogen molecule. The fraction *η*_V_ of electron energy going to vibrational excitation of N_2_(X^1^∑_g_^+^, v) is determined from the corresponding electron energy loss coefficients given by the BOLSIG+ software. The fraction *η*_T_ is determined in the same manner but considering the electron energy loss coefficients corresponding to the excitation of the translational and rotational modes of molecules in air, and the vibrational excitation of O_2_ followed by a fast V-T relaxation. τ_VT_ is the timescale of the V*-*T energy relaxation by molecules and atoms collisions [34]. The V*-*T relaxation of N_2_(X^1^∑_g_^+^, v) molecules on O(^3^P) atoms is described by using the experimental data presented in [73,74,75]. *c*_p_ is the specific heat of the heavy particle of the *Y*_i_ species calculated by assuming that the translational and rotational energies stored per particle are equal to their classical values (= 7/2 *k*_b_ for a diatomic molecule because temperatures are equilibrated in air discharges [34]). *Q*_R_ is the ‘fast’ gas heating rate term due to the electronic- translational (E-T) relaxation energy suggested in [76], to describe observations at moderate values of reduced electric fields. Reactions where energy release is considered for the gas heating term *Q*_R_ in Equation (4) are accompanied by the exothermic energy *ε*_R_ in the right side of the equations in Table 1. The current density *j* of the discharge can be related with the electric field strength in the discharge column by using the Ohm’s law.

The balance equations are solved numerically by a finite-difference explicit method with the second-order approximation in time. Because of the stiffness of the equations (there is a wide range of timescales related with different plasma processes, which reduces the time-step needed for accurate numerical integration), a short time-step for integration (= 1.0 × 10^–10^ s) is used. At the initial time-instant (*t* = 0), it is assumed that the reduced electric field has a value in the range 100–200 Td, intermediate between relatively low *E*/*N* in the column and high *E*/*N* in the cathode part. (A variation of this value inside the above interval has a negligible effect on the calculated discharge parameters [23]). The electron number density for a given current density value is determined by the Ohm’s law. The initial densities of negative ions are estimated using the local balance equations, and the density of positive ions is obtained from the condition of quasi-neutrality. According to [13,14], the preheated air enters the discharge column in a state close to the thermodynamic equilibrium at *T*_0_, hence, the densities of neutral species at *t* = 0 are taken to be close to the equilibrium values at *T*_0_. The initial conditions for Equations (3) and (4) are as follows: *T*_g_(0) = *T*_0_, *ε*_v_(0) = *ε*_v_(*T*_0_). The balance equations are integrated from these initial values up to times of about 10^−3^ s (it was sufficient for the density of each species to converge within an error of about 10^−3^ to its equilibrium value). The gas flow velocity *u* is assumed to vary proportionally to *T*_g_,

(5) $$u=u_{0}\;\frac{T_{g}}{T_{0}}$$

to consider the increase of gas temperature due to gas heating inside the discharge [49]. In [13,14], the current density values are measured by emission spectroscopy of the N_2_ C state, and thus are representative of the discharge region with high electron number density. That is, the model output gives averaged values of the plasma quantities over the discharge axis, but corresponding to the central region of the current–carrying area of the discharge.

## 3. Results and Discussion

**Figure 1:**
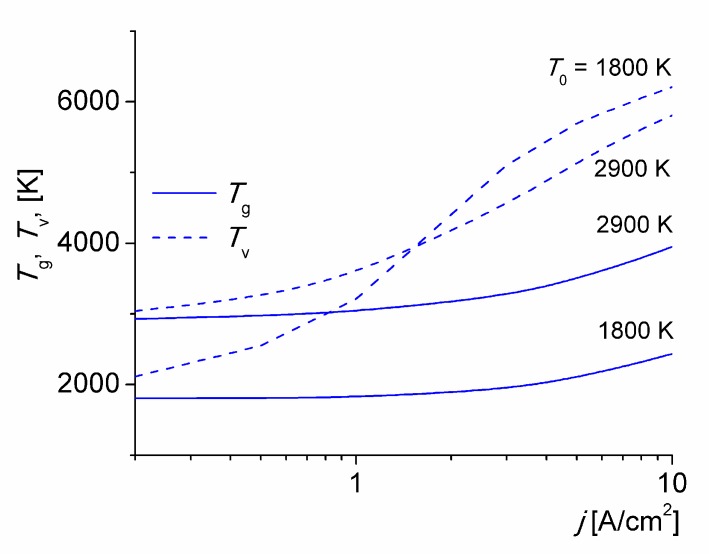
Gas and vibrational temperatures versus the discharge current density for various *T*_0_.

Gas and vibrational temperatures calculated at various values of *T*_0_ versus the discharge current density is given in Figure 1. The gas and vibrational temperatures increase with *j*. For *j* > 1 A/cm^2^ the values of *T*_v_ are larger at smaller *T*_0_, thus increasing the non-equilibrium state of the discharge. This is expected since the rate of V-T energy relaxation increases strongly with the gas temperature. The same trend is shown in [23]. Over the mean-convective transport time (*τ* ≈ 33 μs) the gas at *T*_0_ = 1800 K is heated by about 300 K for *j* = 5 A/cm^2^, due to an incomplete V*-*T energy relaxation. Note under these specific conditions, the timescale of the V*-*T energy relaxation of N_2_(X^1^∑_g_^+^, v) molecules on O(^3^P) atoms collisions is τ_VT_ ≈ 86 μs. The major source of gas heating, however, is the V-T relaxation of nitrogen molecules. The E-T energy relaxation does not play a relevant role under these conditions (i.e., *η*_T_ + *η*_V_ ≈ 1 [15,34]) The *T*_e_ values (not shown) softly decreases while the discharge current density (and gas temperature) increases; with values around 9000 K, typical of this kind of low-current discharges in molecular gases [20,21,22,23,24].

**Figure 2:**
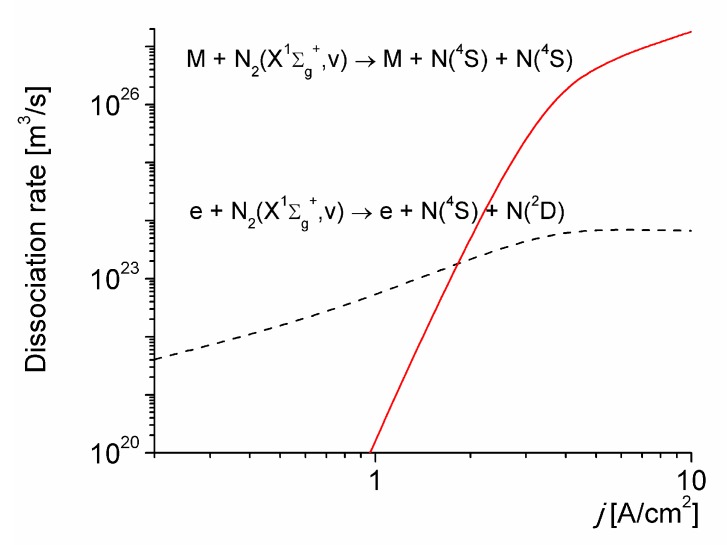
Dissociation rates versus the discharge current density for *T*_0_ = 1800 K.

Figure 2 shows a comparison between the electron-impact dissociation from all the vibrational levels of N_2_(X^1^∑_g_^+^, v), with the thermal dissociation for vibrational excited molecules for *T*_0_ = 1800 K. As it can be seen, the thermal dissociation, stimulated by vibrational non-equilibrium, dominates over the electron-impact dissociation for *j* > 2 A/cm^2^; being the dominant mechanism in the production of N(^4^*S*) atoms under the conditions considered.

**Figure 3:**
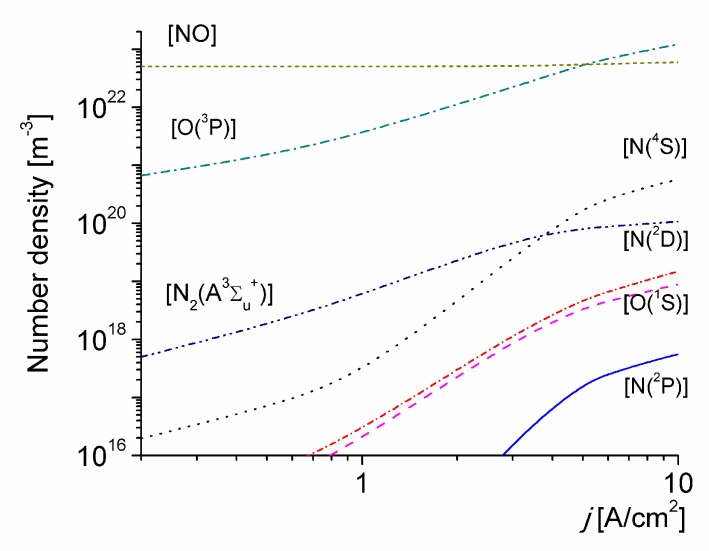
The number density of selected neutral species versus the discharge current density for *T*_0_ = 1800 K.

**Figure 4:**
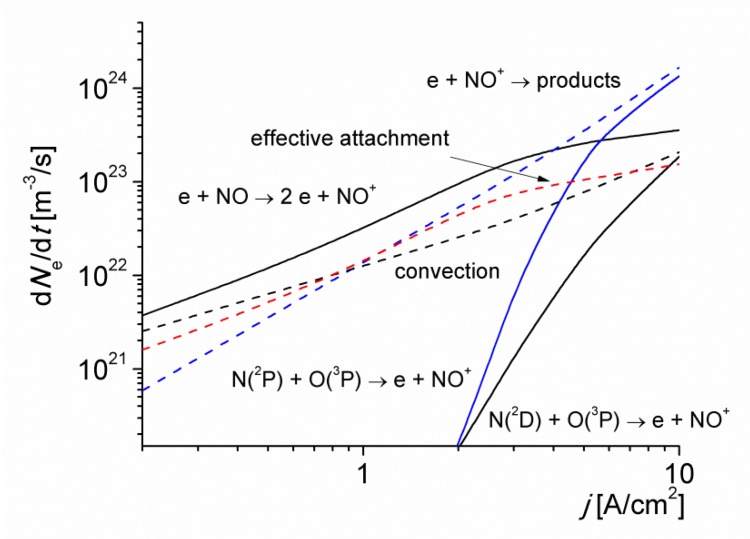
The rates of electron production and loss via various mechanisms versus the discharge current density for *T*_0_ = 1800 K.

The number density of several neutral species versus the discharge current density for *T*_0_ = 1800 K is shown in Figure 3. The density of NO molecules is fairly high under the conditions considered, due to the high-initial gas temperature value. The rather-high vibrational non-equilibrium state of the discharge for high-values of *j* promotes the production of N(^4^S) atoms due to thermal dissociation of N_2_(X^1^∑_g_^+^, v) molecules (the N(^4^S) atoms number density being orders of magnitude higher than the corresponding to the local thermal equilibrium at *T*_g_ [77]), and also leads to a significantly speeds up of the production of N_2_(A^3^∑_u_^+^) molecules by electron-impact excitation from all the vibrational levels of N_2_(X^1^∑_g_^+^, v). The increase of the density of N_2_(A^3^∑_u_^+^) molecules, cause in turn, a rise in the concentration of O(^3^P) atoms because O_2_ molecules are intensively dissociated in the quenching reaction (R44) of N_2_(A^3^∑_u_^+^) molecules. Subsequent quenching of N_2_(A^3^∑_u_^+^) molecules by N(^4^S) leads in turn to the generation of N(^2^P) metastable atoms trough reactions (R50), while the quenching of N_2_(A^3^∑_u_^+^) by O(^3^P) atoms produce both O(^1^S) metastable atoms trough reactions (R48) and N(^2^D) metastable atoms trough reaction (R49). These exited atoms may participate in reactions of associative ionization (R14), (R16) and (R18), as shown in Figure 4.

The rates of electron production and loss via various mechanisms versus the discharge current density for *T*_0_ = 1800 K is shown in Figure 4. At this low-gas temperature value, the electrons are efficiently lost in reactions of dissociative (R84) and three-body (R83) attachment. These processes, however, are balanced to a great extent by the rapid destruction of negative ions in their interaction with NO molecules via the reaction (R89), and with O(^3^P) atoms for current density values higher than 3 A/cm^2^ (see Figure 3), via the reactions (R86) and (R88). The curve labeled as ‘effective’ attachment represents the difference between the rates of attachment (R83) and (R84), and detachment (R85)–(R90) reactions. The convection due to gas flow as well as the electron attachment are the main channels for the loss of electrons at current density values lower than 1 A/cm^2^. Note that the gas flow has practically no direct effect on the motion of electrons. However, due to the coupling between the electrons and ions through the ambipolar field, the electron trajectories correspond to a field distribution in the discharge that takes into account the removal of ions by the gas flow (note that the flow velocity of the gas (≈450 m/s) and the drift velocity of ions in the electric field are comparable [15,16]). For larger *j* values, however, the loss of electrons is dominated by the fast electron-ion recombination reaction (R23). Note that the convective charge transport does not significantly perturbs the local particle balance for *j* > 1 A/cm^2^, when the time of the electron-ion recombination (which is inversely proportional to the electron number density) is smaller than the convective transport time of the gas in the discharge. The role of the processes involving negative ions is less important for higher values of *T*_0_, due to the accumulation of components such as NO and O(^3^P), resulting in an increase in the detachment rate. 

It is seen in Figure 4 that the contribution of NO molecules to ionization processes via reaction (R3) is significant because of their relatively low ionization energy (9.27 eV). This result agrees with the inferences made in [23,24,25]. However, the accumulation of N(^2^P) metastable atoms for current density values higher than 3 A/cm^2^, significantly speeds up the ionization kinetics of the discharge; mainly via the following reactions:

(R9) $$e+N_{2}\left(X\right)\;\to \;e+N_{2}\left(A\right)$$

(R50) $$N_{2}\left(A\right)+N\left({}^{4}S\right)\to \;N_{2}\left(X\right)+N\left({}^{2}P\right)$$

(R18) $$N\left({}^{2}P\right)+O\left({}^{3}P\right)\;\to \;{\mathrm{NO}}^{+}+e$$

the rate coefficient of reaction (R18) is weakly gas temperature dependent (it has no activation barrier), and independent of the reduced electric field. In particular, the charged particle generation is controlled predominantly by reaction (R18) for *j* > 5 A/cm^2^. For higher values of the initial gas temperature of the discharge *T*_0_ (not shown), the following reactions become also important in the gas ionization:

(R49) $$N_{2}\left(A\right)+O\left({}^{3}P\right)\to \;\mathrm{NO}+N\left({}^{2}D\right)$$

(R16) $$N\left({}^{2}D\right)+O\left({}^{3}P\right)\;\to \;{\mathrm{NO}}^{+}+e$$

because the rate coefficient of reaction (R16) is strongly gas temperature dependent (it has a low-activation barrier of 0.38 eV), and independent of the reduced electric field. The associative ionization reaction (R13) (with a high-activation barrier of 2.76 eV), does not play a significant role under the considered conditions due to the low-gas temperature of the discharge.

**Figure 5:**
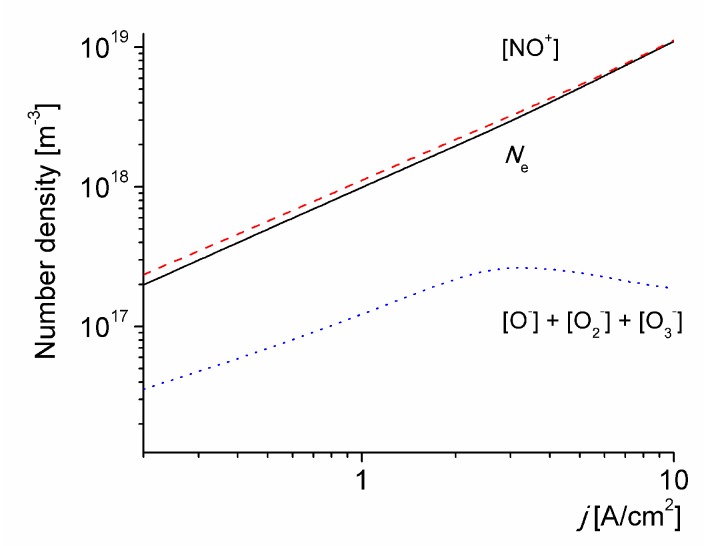
The number density of the charged particles in the discharge versus the discharge current density for *T*_0_ = 1800 K.

Figure 5 shows the number density of the charged particles in the discharge versus the discharge current density for *T*_0_ = 1800 K. For *j* = 2 A/cm^2^ the electron number density is *N*_e_ ≈ 2 × 10^18^ m^-3^, in good agreement with the estimate in [23,24]. It is also observed that the electron number density increases by one order of magnitude when the applied current density is increased by one order of magnitude. This trend is consistent with the estimations in [13]. The total number density of negative ions decreases with *j*, due to the accumulation of O(^3^P) atoms resulting in an increase in the detachment rate.

**Figure 6:**
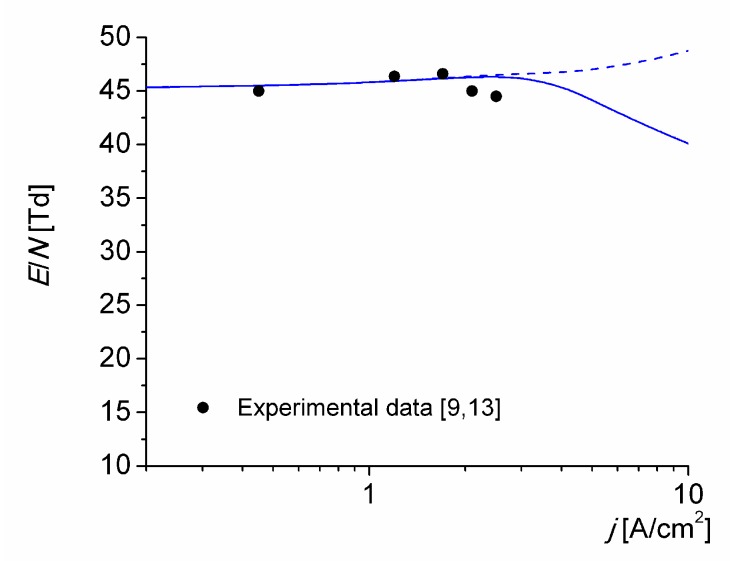
Reduced electric field in the discharge versus the discharge current density for *T*_0_ = 1800 K. Calculations with (solid line) and without (dashed line) associative ionization reactions (R14)–(R16) and (R18).

The reduced electric field (1 Td ≡ 10^−21^ V m^2^) versus the discharge current density is shown in Figure 6, both measured in [9,13] and obtained by simulation for *T*_0_ = 1800 K. Curves are the results of calculations, with (solid line) and without (dashed line) associative ionization reactions (R14)–(R16) and (R18). The reduced electric field given by the solid line follows the changes in the ionization mechanisms shown in Figure 4. It begins to appreciably decrease at current density values higher than 3 A/cm^2^ (i.e., when the reaction (R18) begins to significantly contribute to the ionization kinetics of the discharge) because the electron energy (6.2 eV) required for the excitation of the N_2_(A^3^∑_u_^+^) state is considerably lower than the ionization energy (9.27 eV) of the NO molecules. The calculated reduced electric field values considering the ionization reactions in metastable atomic collisions are seen to be in fairly good agreement with the experimental data over one order of magnitude in current density. 

**Figure 7:**
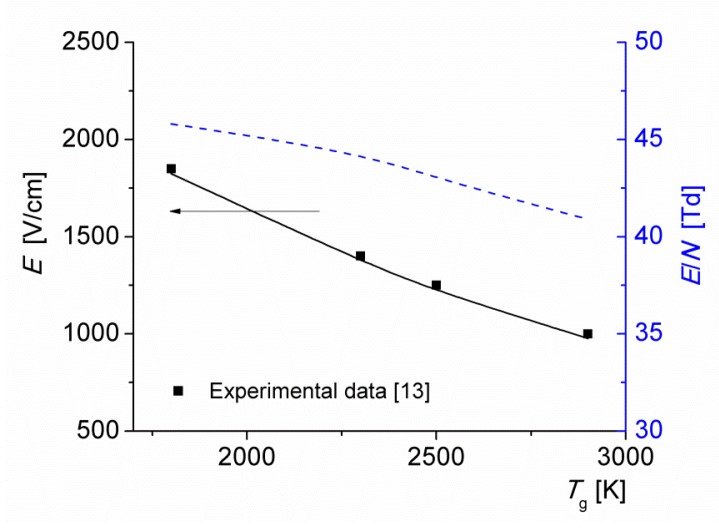
Electric field strength (solid line) and reduced electric field (dashed line) as functions of the initial gas temperature of the discharge *T*_0_ for *j* = 1 A/cm^2^.

Figure 7 shows the average electric field and the reduced electric field versus the initial gas temperature of the discharge *T*_0_ = 1800–2900 K, for a current density *j* = 1 A/cm^2^. The results of calculations are in good agreement with the experimental data [13]. Note that the reduced electric field depends weakly on the gas temperature: as *T*_0_ increases from 1800 to 2900 K, the reduced field decreases from 46 to 41 Td. The decrease is caused by the formation of NO molecules with low–ionization energy (note from Figure 4 that at *j* = 1 A/cm^2^, the main ionization process is via the reaction (R3)). This agrees with the results of calculations performed in [24,25].

## 4. Conclusions

1. A kinetic scheme for non-equilibrium regimes of atmospheric pressure air discharges is developed. An improvement of the model is that it considers associative ionization with the participation of N(^2^D,^2^P) exited atoms.
2. The model is used to simulate the parameters of a glow discharge ignited in a fast longitudinal flow of preheated (*T*_0_ = 1800–2900 K) air. The results adequately describe the dependence of the electric field in the glow discharge on the initial gas temperature.
3. The rather–high vibrational non-equilibrium state of the discharge for high current density values, promotes the production of N(^4^S) atoms due to thermal dissociation of N_2_(X^1^∑_g_^+^, v) molecules, and also leads to a significantly speeds up of the production of N_2_(A^3^∑_u_^+^) molecules by electron-impact excitation from all the vibrational levels of N_2_(X^1^∑_g_^+^, v).
4. For *T*_0_ = 1800 K, the accumulation of N(^2^P) metastable atoms at current density values higher than 3 A/cm^2^, significantly speed up the ionization kinetics of the discharge; mainly via the following reactions:
  $$e+N_{2}\left(X^{1}\Sigma_{g}^{+},\;v\right)\;\to \;e+N_{2}\left(A^{3}\Sigma_{u}^{+}\right),$$
  $$N_{2}\left(A^{3}\Sigma_{u}^{+}\right)+N\left({}^{4}S\right)\to \;N_{2}\left(X^{1}\Sigma_{g}^{+},\;v\right)+N\left({}^{2}P\right),$$
  $$O\left({}^{3}P\right)+N\left({}^{2}P\right)\to \;{\mathrm{NO}}^{+}+e,$$
  accordingly; the reduced electric field begins to appreciably decrease at current densities higher than 3 A/cm^2^, because the electron energy (6.2 eV) required for the excitation of the N_2_(A^3^∑_u_^+^) state is considerably lower than the ionization energy (9.27 eV) of the NO molecules. For higher values of *T*_0_, the following reactions become also important in the charged particles production:
  $$N_{2}\left(A^{3}\Sigma_{u}^{+}\right)+O\left({}^{3}P\right)\to \;\mathrm{NO}+N\left({}^{2}D\right),$$
  $$O\left({}^{3}P\right)+N\left({}^{2}D\right)\to \;{\mathrm{NO}}^{+}+e,$$
  because the reaction between N(^2^D) and O(^3^P) atoms is strongly dependent on the gas temperature.

## Figures and Tables

**Table 1:** List of reactions and rate coefficients and applicable references.

No. *j*	Reaction	Rate Coefficient [m^3^/s or m^6^/s]	Reference
***Electron-Impact Processes***			
R1	e + N_2_(X) → e + e + N_2_^+^	*k*_1_ = *f* (*E*/*N*)	[32,33]
R2	e + O_2_ → e + e + O_2_^+^	*k*_2_ = *f* (*E*/*N*)	[32,33]
R3	e + NO → e + e + NO^+^	*k*_3_ = *f* (*E*/*N*)	[32,33]
R4	e + O(^3^P) → e + e + O^+^	*k*_4_ = *f* (E/*N*)	[32,33]
R5	e + N_2_(X) → e + N_2_*(∆E = 13 eV) e + N(^4^S) + N(^2^D)	*k_5_ = f (E/N)*	[32,33]
R6	e + O_2_ → e + O_2_* (∆E = 6.0 eV) e + O(^3^P) + O(^3^P) + 0.8 eV	*k*_6_ = *f* (*E*/*N*)	[32,33]
R7	e + O_2_ → e + O_2_ (∆E = 8.4 eV) e + O(^3^P) + O(^1^D) + 1.26 eV	*k*_7_ = *f* (*E*/*N*)	[32,33]
R8	e + O_2_ → e + O_2_ (∆E = 9.97 eV) e + O(^3^P) + O(^1^S) + 0.6 eV	*k*_8_ = *f* (*E*/*N*)	[32,33]
R9	e + N_2_(X) → e + N_2_(A)	*k*_9_ = *f* (*E*/*N*)	[32,33]
R10	e + N_2_(X) → e + N_2_(B)	*k*_10_ = *f* (*E*/*N*)	[32,33]
R11	e + N_2_(X) → e + N_2_(a’)	*k*_11_ = *f* (*E*/*N*)	[32,33]
R12	e + N_2_(X) → e + N_2_(C)	*k*_12_ = *f* (*E*/*N*)	[32,33]
***Associative Ionization***			
R13	N(^4^S) + O(^3^P) → NO^+^ + e	*k*_13_ = 5 × 10^−17^ *T*_g_^−0.5^ exp(−32,500/*T*_g_)	[35]
R14	N(^4^S) + O(^1^S) → NO^+^ + e	*k*_14_ = (1–3) × 10^−17^ (*T*_g_/300)^1/6^	[36]
R15	N(^4^S) + O(^1^D) → NO^+^ + e	*k*_15_ = 3.1 × 10^−25^ *T*_g_^0.5^ (9287 + 2 *T*_g_) exp(–9287/*T*_g_)	[37]
R16	N(^2^D) + O(^3^P) → NO^+^ + e	*k*_16_ = 1.3 × 10^−24^ *T*_g_^0.5^ (4411 + 2 *T*_g_) exp(–4411/*T*_g_)	[38,39]
R17	N(^2^D) + N(^2^P) → N_2_^+^ + e	*k*_17_ = 1.9 × 10^−21^ *T*_g_^0.98^ [1 − exp(–3129/*T*_g_)]^−1^	[40]
R18	N(^2^P) + O(^3^P) → NO^+^ + e	*k*_18_ = (1–3) × 10^−17^ (*T*_g_/300)^1/6^	[36]
R19	N(^2^P) + N(^2^P) → N_2_^+^ + e	*k*_19_ = 3.2 × 10^−21^ *T*_g_^0.98^ [1 − exp(−3129/*T*_g_)]^−1^	[40]
***Penning Ionization***			
R20	N_2_(A) + N_2_(a’) → N_2_^+^ + N_2_(X) + e	*k*_20_ = 5 × 10^−17^	[41]
R21	N_2_(a’) + N_2_(a’) → N_2_^+^ + N_2_(X) + e	*k*_21_ = 2 × 10^−16^	[41]
***Dissociative Electron-Ion Recombination***			
R22	e + NO^+^ → N(^4^S) + O(^3^P)	*k*_22_ = 0.05 ×1.5 × 10^−11^ *T*_e_^−0.65^ *k*_22_ = 0.05 ×1.1 × 10^−8^ *T*_e_^−1.5^	[42,43]
			[43,44]
R23	e + NO^+^ → N(^2^D) + O(^3^P)	*k*_23_ = 0.95 × 1.5 × 10^-11^ *T*_e_^-0.65^ *k*_23_ = 0.95 ×1.1 × 10^-8^ *T*_e_^-1.5^	[42,43] [43,44]
R24	e + N_2_^+^ → N(^4^S) + N(^2^D)	*k*_24_ = 0.46 × 2.0 × 10^−13^ (300/*T*_e_)^0.5^	[45,46]
R25	e + N_2_^+^ → N(^4^S) + N(^2^P)	*k*_25_ = 0.08 × 2.0 × 10^−13^ (300/*T*_e_)^0.5^	[45,46]
R26	e + N_2_^+^ → N(^2^D) + N(^2^D)	*k*_26_ = 0.46 × 2.0 × 10^−13^ (300/*T*_e_)^0.5^	[45,46]
R27	e + O_2_^+^ → O(^3^P) + O(^3^P)	*k*_27_ = 0.32 × 2.0 × 10^−13^ (300/*T*_e_)	[45,46]
R28	e + O_2_^+^ → O(^3^P) + O(^1^D)	*k*_28_ = 0.43 × 2.0 × 10^−13^ (300/*T*_e_)	[45,46]
R29	e + O_2_^+^ → O(^1^D) + O(^1^D)	*k*_29_ = 0.20 × 2.0 × 10^−13^ (300/*T*_e_)	[45,46]
R30	e + O_2_^+^ → O(^1^D) + O(^1^S)	*k*_30_ = 0.05 × 2.0 × 10^−13^ (300/*T*_e_)	[45,46]
***Three Body Electron-Ion Recombination***			
R31	e + e + O^+^ → e + O(^3^P)	*k*_31_ = 1.0 × 10^−31^ (300/*T*_e_)^4.5^	[45]
***Thermal Dissociation/Three Body Recombination***			
R32	N_2_(X) + M → N(^4^S) + N(^4^S) + M M = N_2_(X), O_2_, NO	*k*_32_ = 5 × 10^−14^ exp(–113,200/*T*_g_) [1 – exp(–3354/*T*_g_)]	[25]
R33	N_2_(X) + M → N(^4^S) + N(^4^S) + M M = N(^4^S), O(^3^P)	*k*_33_ = 1.1 × 10^−^^13^ exp(–113,200/*T*_g_) [1 − exp(−3354/*T*_g_)]	[25]
R34	N(^4^S) + N(^4^S) + M → N_2_(X) + M M = N_2_(X), O_2_, NO, O(^3^P), N(^4^S)	*k*_34_ = 8.27 × 10^−46^ exp(500/*T*_g_)	[25]
R35	O_2_(X) + M → O(^3^P) + O(^3^P) + M M = O_2_	*k*_35_ = 3.7 × 10^−14^ exp(–59,380/*T*_g_) [1 − exp(−2240/*T*_g_)]	[25]
R36	O_2_(X) + M → O(^3^P) + O(^3^P) + M M = O(^3^P)	*k*_36_ = 1.3 × 10^−13^ exp(–59,380/*T*_g_) [1 − exp(−2240/*T*_g_)]	[25]
R37	O_2_(X) + M → O(^3^P) + O(^3^P) + M M = N_2_(X), N(^4^S), NO	*k*_37_ = 9.3 × 10^−15^ exp(−59,380/*T*_g_) [1 − exp(−2240/*T*_g_)]	[25]
R38	O(^3^P) + O(^3^P) + M → O_2_(X) + M M = N_2_(X)	*k*_38_ = 2.76 × 10^−46^ exp(720/*T*_g_)	[25]
R39	O(^3^P) + O(^3^P) + M → O_2_(X) + M M = O_2_	*k*_39_ = 2.45 × 10^−43^ *T*_g_^−0.63^	[25]
R40	O(^3^P) + O(^3^P) + M → O_2_(X) + M M = O(^3^P)	*k*_40_ = 8.8 × 10^−43^ *T*_g_^−0.63^	[25]
R41	NO + M → N(^4^S) + O(^3^P) + M M = N_2_(X), O_2_	*k*_41_ = 8.7 × 10^−15^ exp(−76,000/*T*_g_)	[25]
R42	NO + M → N(^4^S) + O(^3^P) + M M = O(^3^P), NO	*k_4_*_2_ = 1.7 × 10^−13^ exp(−76,000/*T*_g_)	[25]
R43	N(^4^S) + O(^3^P) + M → NO(X) + M M = N_2_(X), O_2_,NO,O(^3^P)	*k*_43_ = 1.76 × 10^−43^ *T*_g_^−0.5^	[25]
***Chemical Reactions***			
R44	N_2_(A) + O_2_ → N_2_(X) + 2 O(^3^P) + 1.1 eV	*k*_44_ = 1.7 × 10^−18^	[47]
R45	N_2_(A) + O_2_ → N_2_(X) + O_2_(b)	*k*_45_ = 7.5 × 10^−19^	[47]
R46	N_2_(A) + N_2_(A) → N_2_(X) + N_2_(B)	*k*_46_ = 7.7 × 10^−17^	[48]
R47	N_2_(A) + N_2_(A) → N_2_(X) + N_2_(C)	*k*_47_ = 1.6 × 10^−16^	[48]
R48	N_2_(A) + O(^3^P) → N_2_(X) + O(^1^S)	*k*_48_ = 2.1 × 10^−17^	[45]
R49	N_2_(A) + O(^3^P) → NO + N(^2^D)	*k*_49_ = 7.0 × 10^−18^	[45]
R50	N_2_(A) + N(^4^S) → N_2_(X) + N(^2^P)	*k*_50_ = 5.0 × 10^−17^	[49]
R51	N_2_(A) + NO → N_2_(X) + NO	*k*_51_ = 6.4 × 10^−17^	[47]
R52	N_2_(B) + O_2_ → N_2_(X) + 2 O(^3^P)	*k*_52_ = 3.0 × 10^−16^	[45]
R53	N_2_(B) + N_2_(X) → N_2_(X) + N_2_(A)	*k*_53_ = 1.0 × 10^−17^	[49]
R54	N_2_(a’) + O_2_ → N_2_(X) + O(^3^P) + O(^1^D) + 1.4 eV	*k*_54_ = 2.8 × 10^−17^	[45]
R55	N_2_(a’) + N_2_(X) → N_2_(X) + N_2_(B)	*k*_55_ = 2.0 × 10^−19^	[45]
R56	N_2_(a’) + O(^3^P) → NO + N(^2^D)	*k*_56_ = 3.0 × 10^−16^	[50]
R57	N_2_(a’) + NO → N(4S) + O(^3^P) + N_2_(X)	*k*_57_ = 3.6 × 10^−16^	[51]
R58	N_2_(C) + O_2_ → N_2_(X) + 2 O(^3^P)	*k*_58_ = 2.5 × 10^−16^	[52]
R59	N_2_(C) + N_2_(X) → N_2_(X) + N_2_(B)	*k*_59_ = 1.0 × 10^−17^	[52]
R60	N_2_(C) → N_2_(B) + *hυ*	*k*_60_ = 2.4 × 10^7^ s^−1^	[45]
R61	N(^4^S) + NO → O(^3^P) + N_2_(X)	*k*_61_ = 1.0 × 10^−18^ *T*_g_^0.5^	[45]
R62	N(^4^S) + O_2_ → O(^3^P) + NO	*k*_62_ = 1.1 × 10^−20^ *T*_g_ exp(−3150/*T*_g_)	[45]
R63	N(^2^D) + N_2_(X) → N(^4^S) + N_2_(X)	*k*_63_ = 1.7 × 10^−20^	[47]
R64	N(^2^D) + O(^3^P) → N(^4^S) + O(^3^P)	*k*_64_ = 1.4 × 10^−^^18^	[47]
R65	N(^2^D) + O_2_ → NO + O(^3^P)	*k*_65_ = 2.4 × 10^−^^18^ exp(−185/*T*_g_)	[47]
R66	N(^2^D) + O_2_ → NO + O(^1^D)	*k*_66_ = 7.3 × 10^−^^18^ exp(−185/*T*_g_)	[47]
R67	N(^2^D) + NO → N_2_(X) + O(^1^S)	*k*_67_ = 6.0 × 10^−^^17^	[47]
R68	N(^2^P) + N(^4^S) → N(^2^D) + N(^4^S)	*k*_68_ = 1.8 × 10^−^^18^	[45]
R69	N(^2^P) + O(^3^P) → N(^2^D) + O(^3^P)	*k*_69_ = 1.0 × 10^−^^18^	[49]
R70	N(^2^P) + O_2_ → NO + O(^3^P)	*k*_70_ = 2.5 × 10^−^^18^	[47]
R71	N(^2^P) + NO → N_2_(X) + O(^3^P)	*k*_71_ = 2.9 × 10^−^^17^	[47]
R72	O(^3^P) + N_2_(X) → N(^4^S) + NO	*k*_72_ = 1.3 × 10^−^^16^ exp(−38,000/*T*_g_)	[25]
R73	O(^3^P) + NO → N(^4^S) + O_2_	*k*_73_ = 2.5 × 10^−^^21^ *T*_g_ exp(−19,500/*T*_g_)	[25]
R74	O(^1^D) + O(^3^P) → O(^3^P) + O(^3^P)	*k*_74_ = 8.0 × 10^−^^18^	[45]
R75	O(^1^D) + O_2_ → O(^3^P) + O_2_(b)	*k*_75_ = 3.2 × 10^−^^17^ exp(67/*T*_g_)	[45]
R76	O(^1^D) + N_2_(X) → O(^3^P) + N_2_(X) + 1.4 eV	*k*_76_ = 1.8 × 10^−^^17^ exp(107/*T*_g_)	[45]
R77	O(^1^S) + O(^3^P) → O(^1^D) + O(^3^P)	*k*_77_ = 5.0 × 10^−^^17^ exp(−301/*T*_g_)	[45]
R78	O(^1^S) + O_2_ → O_2_ + O(^3^P)	*k*_78_ = 3.0 × 10^−^^18^ exp(−850/*T*_g_)	[45]
R79	O(^1^S) + O_2_ → O_2_ + O(^1^D)	*k*_79_ = 1.3 × 10^−^^18^ exp(−850/*T*_g_)	[45]
R80	O(^1^S) + N(^4^S) → O(^3^P) + N(^2^P)	*k*_80_ = 1.0 × 10^−^^18^	[49]
R81	O(^1^S) + NO → O(^3^P) + NO	*k*_81_ = 1.8 × 10^−^^16^	[45]
R82	O(^1^S) + NO → O(^1^D) + NO	*k*_82_ = 3.2 × 10^−^^16^	[45]
***Electron Attachment and Detachment***			
R83	e + O_2_ + O_2_ → O_2_^–^ + O_2_	*k*_83_ = 1.4 × 10^−41^ (300/*T*_e_) exp(−660/*T*_g_) exp[700 (*T*_e_ − *T*_g_)/(*T*_e_ *T*_g_)]	[45]
R84	e + O_2_ → O^–^ + O(^3^P)	*k*_84_ = *f*(*E*/*N*)	[32,33]
R85	O_2_^–^ + O_2_ → O_2_ + O_2_ + e	*k*_85_ = 2.7 × 10^−^^16^ (*T*_g_/300)^0.5^ exp(–5590/*T*_g_)	[45]
R86	O_2_^–^ + O(^3^P) → O_3_ + e	*k*_86_ = 1.5 × 10^−^^16^	[45]
R87	O^–^ + N_2_(X) → N_2_O + e	*k*_87_ = 9.0 × 10^−^^19^	[45]
R88	O^–^ + O(^3^P) → O_2_ + e	*k*_88_ = 5.0 × 10^−^^16^	[45]
R89	O^–^ + NO → NO_2_ + e	*k*_89_ = 2.6 × 10^−^^16^	[45]
R90	O_3_^–^ + O(^3^P) → O_2_ + O_2_ + e	*k*_90_ = 3.0 × 10^−^^16^	[45]
***Ion Conversion***			
R91	O^–^ + O_2_(X) + M → O_3_^–^ + M M = N_2_(X),O_2_	*k*_91_ = 1.1 × 10^−^^42^ (300/*T*_g_)	[45]
R92	O^+^ + N_2_(X) → NO^+^ + N(^4^S)	*k*_92_ = (1.5–2.0 × 10^−^^3^ *T*_g_ + 9.56 × 10^−^^7^ *T*_g_^2^) × 10^−^^18^	[49]
R93	N_2_^+^ + O_2_(X) → N_2_(X) + O_2_^+^	*k*_93_ = 6 × 10^−^^17^ (300/*T*_g_)^0.5^	[45]
R94	N_2_^+^ + O(^3^P) → N_2_(X) + O^+^	*k*_94_ = 1.0 × 10^−^^17^ (300/*T*_g_)^0.2^	[45]
R95	N_2_^+^ + O(^3^P) → NO^+^ + N(^4^S)	*k*_95_ = 0.95 × 1.3 × 10^−^^16^ (300/*T*_g_)^0.5^	[45,53]
R96	N_2_^+^ + O(^3^P) → NO^+^ + N(^2^D)	*k*_96_ = 0.05 × 1.3 × 10^−^^16^ (300/*T*_g_)^0.5^	[45,53]
R97	O_2_^+^ + NO → NO^+^ + O_2_	*k*_97_ = 6.3 × 10^−^^16^	[49]
***Ion-Ion Recombination***			
R98	X^–^ + Y^+^ → X + Y X^–^ = O^–^, O_2_^–^, O_3_^–^ Y^+^ = N_2_^+^, O_2_^+^, NO^+^, O^+^	*k*_98_ = 2.0 × 10^−^^13^ (300/*T*_g_)^0.5^	[45]

*T*_e_ and *T*_g_ units are in Kelvin.

## References

[1] André P., Barinov Y.A., Faure G., Shkol’nik S.M. (2018). Characteristics of discharge with liquid nonmetallic cathode burning in air flow. J. Phys. D Appl. Phys..

[2] Staack D., Farouk B., Gutsol A., Fridman A. (2005). Characterization of a dc atmospheric pressure normal glow discharge. Plasma Sources Sci. Technol..

[3] Staack D., Farouk B., Gutsol A., Fridman A. (2008). DC normal glow discharges in atmospheric pressure atomic and molecular gases. Plasma Sources Sci. Technol..

[4] Verreycken T., Schram D.C., Leys C., Bruggeman P. (2010). Spectroscopic study of an atmospheric pressure dc glow discharge with a water electrode in atomic and molecular gases. Plasma Sources Sci. Technol..

[5] Stark R.H., Schoenbach K.H. (1999). Direct current glow discharges in atmospheric air. Appl. Phys. Lett..

[6] Leipold F., Stark R.H., El–Habachi A., Schoenbach K.H. (2000). Electron density measurements in an atmospheric pressure air plasma by means of infrared heterodyne interferometry. J. Phys. D Appl. Phys..

[7] Duten X., Packan D., Yu L., Laux C.O., Kruger C.H. (2002). DC and Pulsed Glow Discharges in Atmospheric Pressure Air and Nitrogen. IEEE Trans. Plasma Sci..

[8] Prevosto L., Kelly H., Mancinelli B., Chamorro J.C., Cejas E. (2015). On the physical processes ruling an atmospheric pressure air glow discharge operating in an intermediate current regime. Phys. Plasmas.

[9] Machala Z., Laux C.O., Kruger C.H., Candler G.V. Atmospheric Air and Nitrogen DC Glow Discharges with Thermionic Cathodes and Swirl Flow. Proceedings of the 42nd AIAA Aerospace Sciences Meeting and Exhibit.

[10] Machala Z., Marode E., Laux C.O., Kruger C.H.J. (2004). DC Glow Discharges in Atmospheric Pressure Air. Adv. Oxid. Technol..

[11] Arkhipenko V.I., Kirillov A.A., Safronau Y.A., Simonchika L.V., Zgirouski S.M. (2012). Plasma non-equilibrium of the DC normal glow discharges in atmospheric pressure atomic and molecular gases. Eur. Phys. J. D.

[12] Akishev Y., Goossens O., Callebaut T., Leys C., Napartovich A., Trushkin N. (2001). The influence of electrode geometry and gas flow on corona-to-glow and glow-to-spark threshold currents in air. J. Phys. D Appl. Phys..

[13] Yu L., Laux C.O., Packan D.M., Kruger C.H.J. (2002). Direct-current glow discharges in atmospheric pressure air plasmas. Appl. Phys..

[14] Laux C.O., Yu L., Packan D.M., Gessman R.J., Pierrot L., Kruger C.H., Zare R.N. Ionization Mechanisms in Two-Temperature Air Plasmas. Proceedings of the 30th Plasmadynamic and Lasers Conference.

[15] Raizer Y.P. (1991). Gas Discharge Physics.

[16] Velikhov E.P., Golubev V.S., Pashkin S.V. (1982). Glow discharge in a gas flow. Sov. Phys. Usp..

[17] Adamovich I., Baalrud S.D., Bogaerts A., Bruggeman P.J., Cappelli M., Colombo V., Czarnetzki U., Ebert U., Eden J.G., Favia P. (2017). The 2017 Plasma Roadmap: Low temperature plasma science and technology. J. Phys. D Appl. Phys..

[18] Naidis G.V. (1999). Simulation of streamer-to-spark transition in short non-uniform air gaps. J. Phys. D Appl. Phys..

[19] Naidis G.V. (2005). Dynamics of streamer breakdown of short non-uniform air gaps. J. Phys. D Appl. Phys..

[20] Benilov M.S., Naidis G.V. (2003). Modelling of low-current discharges in atmospheric-pressure air taking account of non-equilibrium effects. J. Phys. D Appl. Phys..

[21] Naidis G.V. (2007). Simulation of convection-stabilized low-current glow and arc discharges in atmospheric-pressure air. Plasma Sources Sci. Technol..

[22] Xaubet M., Giuliani L., Grondona D., Minotti F. (2017). Experimental and theoretical study of an atmospheric air plasma-jet. Phys. Plasmas.

[23] Benilov M.S., Naidis G.V. (2005). Modelling of discharges in a flow of preheated air. Plasma Sources Sci. Technol..

[24] Popov N.A. (2006). Simulations of a longitudinal glow discharge in a hot air flow at atmospheric pressure. Plasma Phys. Rep..

[25] Aleksandrov N.L., Bazelyan E.M., Kochetov I.V., Dyatko N.A. (1997). The ionization kinetics and electric field in the leader channel in long air gaps. J. Phys. D Appl. Phys..

[26] Popov N.A. (2003). Formation and Development of a Leader Channel in Air. Plasma Phys. Rep..

[27] da Silva C.L., Pasko V.P. (2013). Dynamics of streamer-to-leader transition at reduced air densities and its implications for propagation of lightning leaders and gigantic jets. J. Geophys. Res..

[28] Aleksandrov N.L., Bazelyan E.M. (1999). Ionization processes in spark discharge plasmas. Plasma Sources Sci. Technol..

[29] Aleksandrov N.L., Bazelyan E.M.J. (1998). The mechanism of re-breakdown within a post-arc channel in long non-uniform air gaps. Phys. D Appl. Phys..

[30] Aleksandrov N.L., Bazelyan E.M., Konchakov A.M. (2001). Plasma Parameters in the Channel of a Long Leader in Air. Plasma Phys. Rep..

[31] Mankelevich Y.A., Pal A.F., Popov N.A., Rakhimova T.V., Filippov A.V. (2001). Current Dynamics and Mechanisms for the Instability of a Non-Self-Sustained Glow Discharge in Nitrogen. Plasma Phys. Rep..

[32] Hagelaar G.J.M., Pitchford L.C. (2005). Solving the Boltzmann equation to obtain electron transport coefficients and rate coefficients for fluid models. Plasma Sources Sci. Technol..

[33] SIGLO Database. http://www.lxcat.laplace.univ-tlse.fr.

[34] Capitelli M., Ferreira C.M., Gordiets B.F., Osipov A.I. (2000). Plasma Kinetics in Atmospheric Gases.

[35] Lin S.C., Teare J.D. (1963). Rate of Ionization behind Shock Waves in Air. II. Theoretical Interpretations. Phys. Fluids.

[36] Chernyi G.G., Losev S.A., Macheret S.O., Potapkin B.V. (2002). Physical and Chemical Processes in Gas Dynamics: Cross Sections and Rate Constants Vol. 1.

[37] Le Padellec A. (2005). Partial Near Threshold Cross Sections for the Associative Ionization to Form CO^+^, NO^+^ and O_2_^+^. Phys. Scr..

[38] Golubkov G.V., Ozerov G.K. (2014). The Near-Threshold Associative Ionization N(^2^D) + O(^3^P) → NO(X^1^Σ^+^) + e^−^ Reaction. Doklady Phys..

[39] Ringer G., Gentry W.R. (1979). A merged molecular beam study of the endoergic associative ionization reaction N(^2^D)+O(^3^P) →NO^+^+e*^−^*. J. Chem. Phys..

[40] Matveyev A.A., Silakov V.P. (1999). Theoretical study of the role of ultraviolet radiation of the non-equilibrium plasma in the dynamics of the microwave discharge in molecular nitrogen. Plasma Sources Sci. Technol..

[41] Brunet H., Roca Serra J. (1985). Model for a glow discharge in flowing nitrogen. J. Appl. Phys..

[42] Park C. A Review of Reaction Rates in High Temperature Air. Proceedings of the 24th Thermophysics Conference.

[43] Hellberg F., Rosén S., Thomas R., Neau A., Larsson M., Petrignani P., van der Zande W.J. (2003). Dissociative recombination of NO^+^: Dynamics of the X^1^ Σ_+_ and a^3^ Σ_+_ electronic states. J. Chem. Phys..

[44] Kang S.W., Jones W.L., Dunn M.G. (1973). Theoretical and Measured Electron-Density Distributions at High Altitudes. AIAA J..

[45] Kossyi I.A., Kostinsky A.Y., Matveyev A.A., Silakov V.P. (1992). Kinetic scheme of the non-equilibrium discharge in nitrogen-oxygen mixtures. Plasma Sources Sci. Technol..

[46] Florescu A.I., Mitchell J.B.A. (2006). Dissociative recombination. Phys. Rep..

[47] Herron J.T. (1999). Evaluated Chemical Kinetics Data for Reactions of N(^2^D), N(^2^P), and N_2_(A^3^Σ_u_^+^) in the Gas Phase. J. Phys. Chem. Ref. Data.

[48] Piper L.G. (1988). Statetostate N_2_(A^3^Σ_u_^+^) energy pooling reactions. II. The formation and quenching of N_2_(B^3^Πg, v′=1–12). J. Chem. Phys..

[49] Gordiets B.F., Ferreira C.M., Guerra V.L., Loureiro J.M.A.H., Nahorny J., Pagnon D., Touzeau M., Vialle M. (1995). Kinetic Model of a Low–Pressure N_2_–O_2_ Flowing Glow Discharge. IEEE Trans. Plasma Sci..

[50] Shkurenkov I., Burnette D., Lempert W.R., Adamovich I.V. (2014). Kinetics of excited states and radicals in a nanosecond pulse discharge and afterglow in nitrogen and air. Plasma Sources Sci. Technol..

[51] Piper L.G. (1987). Quenching rate coefficients for N_2_(a′^1^Σ_u_^−^). J. Chem. Phys..

[52] Pancheshnyi S.V., Starikovskaia S.M., Starikovskii A.Y. (2000). Collisional deactivation of N_2_(C^3^Π_u_, v = 0,1,2,3) states by N_2_, O_2_, H_2_ and H_2_O molecules. Chem. Phys..

[53] Siskind D.E., Barth D.A., Cleary D.D. (1990). The Possible Effect of Solar Soft X Rays on Thermospheric N itric Oxide. J. Geophys. Res..

[54] Piper L.G. (1993). The reactions of N(^2^P) with O_2_ and O. J. Chem. Phys..

[55] Capitelli M., Colonna G., D’Ammando G., Laporta V., Laricchiuta A. (2014). Nonequilibrium dissociation mechanisms in low temperature nitrogen and carbon monoxide plasmas. Chem. Phys..

[56] Capitelli M., Colonna G., D’Ammando G., Laporta V., Laricchiuta A. (2013). The role of electron scattering with vibrationally excited nitrogen molecules on non-equilibrium plasma kinetics. Phys. Plasmas.

[57] Pietanza L.D., Colonna G., D’Ammando G., Laricchiuta A., Capitelli M. (2015). Vibrational excitation and dissociation mechanisms of CO2 under non-equilibrium discharge and post-discharge conditions. Plasma Sources Sci. Technol..

[58] Park C. Rate Parameters for Electonic Excitation of Diatomic Molecules 1. Electon-Impact Processes. Proceedings of the 46th AIAA Aerospace Sciences Meeting and Exhibit.

[59] Pietanza L.D., Colonna G., D’Ammando G., Laricchiuta A., Capitelli M. (2016). Non equilibrium vibrational assisted dissociation and ionization mechanisms in cold CO2 plasmas. Chem. Phys..

[60] Loureiro J., Ferreira C.M. (1986). Coupled electron energy and vibrational distribution functions in stationary N2 discharges. J. Phys. D Appl. Phys..

[61] Thorsteinsson E.G., Gudmundsson J.T. (2009). A global (volume averaged) model of a nitrogen discharge: I. Steady state. Plasma Sources Sci. Technol..

[62] Dyatko N.A., Kochetov I.V., Napartovich A.P. (2002). Electron Temperature in Nitrogen Afterglow: Dependence of Theoretical Results on the Adopted Set of Cross Sections and on the Type of Molecular Distribution over Vibrational Levels. Plasma Phys. Rep..

[63] Macheret S.O., Rich J.W. (1993). Nonequilibrium dissociation rates behind strong shock waves: Classical model. Chem. Phys..

[64] Fridman A.A., Kennedy L.A. (2004). Plasma Physics and Engineering.

[65] da Silva M.L., Guerra V., Loureiro J. (2007). Two-temperature models for nitrogen dissociation. Chem. Phys..

[66] Dimokatis P.E. (2000). The mixing transition in turbulent flows. J. Fluid Mech..

[67] Komuro A., Ono R., Oda T. (2010). Kinetic model of vibrational relaxation in a humid-air pulsed corona discharge. Plasma Sources Sci. Technol..

[68] D’Angola A., Colonna G., Bonomo A., Bruno D., Laricchiuta A., Capitelli M. (2012). A phenomenological approach for the transport properties of air plasmas. Eur. Phys. J. D.

[69] Hurlbatt A., Gibson A.R., Schroter S., Bredin J., Foote A.P.S., Grondein P., O’Connell D., Gans T. (2017). Concepts, Capabilities, and Limitations of Global Models: A Review. Plasma Process. Polym..

[70] Akishev Y., Grushin M., Karalnik V., Petryakov A., Trushkin N. (2010). On basic processes sustaining constricted glow discharge in longitudinal N_2_ flow at atmospheric pressure. J. Phys. D Appl. Phys..

[71] Boeuf J.P., Kunhardt E.E. (1986). Energy balance in a nonequilibrium weakly ionized nitrogen discharge. J. Appl. Phys..

[72] Prevosto L., Kelly H., Mancinelli B., Chamorro J.C. (2015). On the Gas Heating Mechanism for the Fast Anode Arc Reattachment in a Nontransferred Arc Plasma Torch Operating with Nitrogen Gas in the Restrike Mode. Plasma Chem. Plasma Process..

[73] Breshears W.D., Bird P.F. (1968). Effect of Oxygen Atoms on the Vibrational Relaxation of Nitrogen. J. Chem. Phys..

[74] Eckstrom D.J. (1973). Vibrational relaxation of shockheated N_2_ by atomic oxygen using the ir tracer method. J. Chem. Phys..

[75] McNeal R.J., Whitson M.E., Cooke G.R. (1974). Temperature Dependence of the Quenching of Vibrationally Excited Nitrogen by Atomic Oxygen. J. Geophys. Res..

[76] Popov N.A. (2001). Investigation of the Mechanism for Rapid Heating of Nitrogen and Air in Gas Discharges. Plasma Phys. Rep..

[77] Gleizes A., Gonzalez J.J., Freton P. (2005). Thermal plasma modelling. J. Phys. D Appl. Phys..

